# Remdesivir therapy causing bradycardia in COVID-19 patients: Two case reports

**DOI:** 10.1016/j.idcr.2021.e01254

**Published:** 2021-08-12

**Authors:** Alaaeldin Abdelmajid, Wala Osman, Huda Musa, Hisham Elhiday, Waqar Munir, Muna A. Al.Maslamani, Eman Zeyad Elmekaty

**Affiliations:** a Communicable Diseases Center, Hamad Medical Corporation, Doha, Qatar; b Division of Infectious Diseases, Department of Medicine, Hamad Medical Corporation, Doha, Qatar; c Internal Medicine Department, Hamad Medical Corporation, Doha, Qatar

**Keywords:** Remdesivir, COVID-19, Bradycardia, Viral pneumonia, SARS-CoV-2

## Abstract

The coronavirus disease 2019 (COVID-19) pandemic has been an enormous public health challenge. The pursuit for an effective therapy led to the use of the antiviral drug Remdesivir for hospitalized patients with severe COVID-19 pneumonia. We reported two cases of patients with severe COVID-19 pneumonia and worsening oxygen requirements. Both patients developed sinus bradycardia following the initiation of Remdesivir therapy and reverted after stopping it. One of the patients developed QTc interval prolongation and required intensive care unit admission. The proposed mechanism for Remdesivir-induced bradycardia and cardiac toxicity could be due to the intrinsic electrophysiological properties and the effect on the AV node; yet, further large observational studies are warranted for better understanding and correlation of Remdesivir with cardiac adverse events. Till then, healthcare providers need to be alert of this potential adverse event and to monitor their COVID-19 patients closely while on Remdesivir therapy.

## Introduction

Remdesivir is a prodrug of a cyano-adenosine nucleoside analog [1]. It exserts its antiviral activity through incorporation into SARS-CoV’s RNA chains, leading to chain termination and inhibition of viral replication [2]. To maximize the likelihood of Remdesivir benefit, it should be initiated as early as possible during the viral replication phase of the pathogenesis course of COVID-19 [3], [4], [5], [6]. Treatment of COVID-19 pneumonia using Remdesivir therapy was evaluated in randomized clinical trials of patients with severe COVID-19 infection and was shown to have faster time to recovery when compared to placebo [7], [8]. In clinical trials, Remdesivir adverse event profile has been favorable overall. The most commonly reported adverse events were increased liver aminotransferases, hypersensitivity reactions, nausea, and hypokalemia [7], [8]. Infusion-related reactions have been also reported where patients may experience angioedema, bradycardia, hypotension, and hypoxia [9].

More than 2000 patients were treated with Remdesivir therapy in Qatar since the start of the pandemic. In this case report, we described a probable bradycardia adverse event related to Remdesivir therapy which was rarely described in literature.

### Case presentation 1

A 55-years-old male patient admitted to the hospital after his diagnosis with COVID-19 infection with COVID-19 PCR CT value of 26. He is known to have dyslipidemia on rosuvastatin 20 mg once daily, with no other significant medical comorbidities. He is active smoker (once weekly) and does not have known allergies to drugs.

Upon admission, patient was complaining of body pain and subjective fever. His vital signs were showing desaturation (oxygen saturation of 91% on room air), so he was started on 2 litres oxygen through nasal cannula). his temperature was 36.9 °C, respiratory rate 18–20, heart rate 61, and blood pressure 105/63 mmHg.

**Fig. 1:**
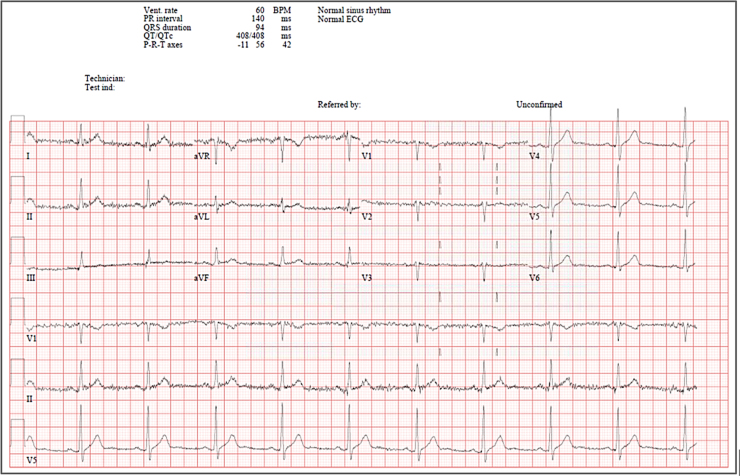
Baseline electrocardiogram for patient case #1 before starting Remdesivir therapy.

Chest X-ray done upon admission was showing bilateral pulmonary opacity and consolidation and his baseline electrocardiogram (ECG) was normal (Fig. 1).

His lab results revealed the following: white blood cell (WBC) 3.2, D-Dimer 0.63, Alanine transaminase (ALT) 53 U/L, Aspartate transaminase (AST) 51 U/L, C-reactive protein (CRP) 47 mg/dl (0–5 mg/dl), ferritin 496 ng/ml, and his renal function was normal (serum creatinine 90 μmol/l, estimated Glomerular Filtration Rate (eGFR) 81 ml/min/1.73 m^2^). Patient was started on Favipiravir, symptomatic treatment, dexamethasone, and enoxaparin for VTE prophylaxis.

**Fig. 2:**
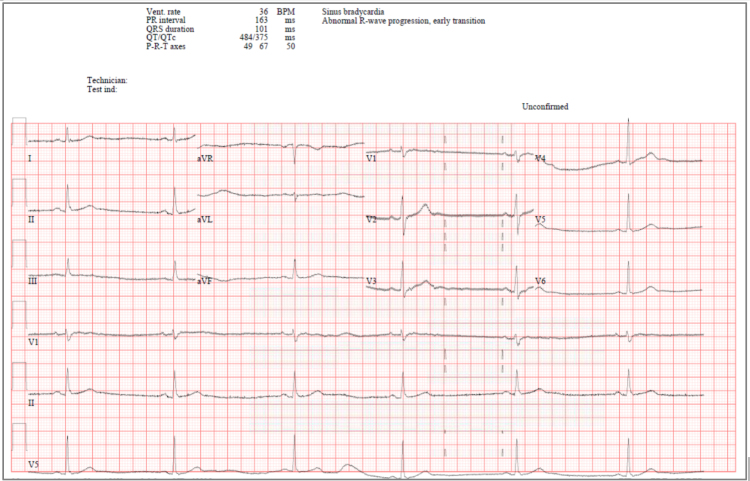
Electrocardiogram for patient case #1 few days after starting Remdesivir therapy.

Few days later, Favipiravir was switched to Remdesivir 200 mg IV once then 100 mg IV from day 2 to day 5. Patient took four doses of Remdesivir, then he started having bradycardia with heart rate of 55–58. His bradycardia continued to worsen reaching 31 beat/minute, so rapid response team was activated to escalate the patient care and management. Patient was assessed closely and his heart rate at that time was 33, blood pressure 112/70 mmHg, oxygen saturation 94%. His electrolytes were within normal range with sodium 144, potassium 4.1, and calcium 2.1. His renal and liver functions after Remdesivir therapy remained within normal ranges, except for slight elevation in ALT levels (ALT 131 U/L, AST 57 U/L, serum creatinine 91 μmol/l, eGFR 80 ml/min/1.73 m^2^). His ECG was showing sinus bradycardia with no dynamic changes (Fig. 2).

**Fig. 3:**
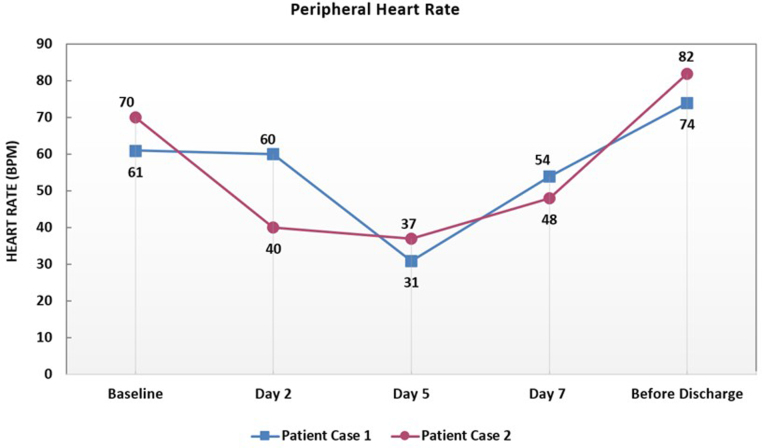
Graphical illustration of the minimum peripheral heart rate values over the course of Remdesivir therapy for both patients. BPM: beat per minute.

Furthermore, thyroid function test was sent to rule out cardiac changes due to thyroid function abnormalities and the results were normal as well with thyroid stimulating hormone (TSH) of 0.4. ProBNP and serial troponin were sent, and the results were within normal range. Therefore, Remdesivir was stopped on day 5 of therapy and the heart rate started to return to normal values the following days and the ECG was normal (Fig. 3).

The patient became asymptomatic and vitally stable on room air and maintaining oxygen saturation of 96–99%. Repeated COVID-19 PCR was negative, and the patient was discharged home in a stable condition.

### Case presentation 2

A 54-years-old woman with no past medical history, presented to the emergency department complaining of fever, shortness of breath, dry cough, and body pain for 7 days. She was tested positive for COVID-19 after a contact with a positive case in her family. She is not a smoker or alcohol drinker and she has positive family history of breast cancer, diabetes mellitus, hypertension, and deep vein thrombosis.

**Fig. 4:**
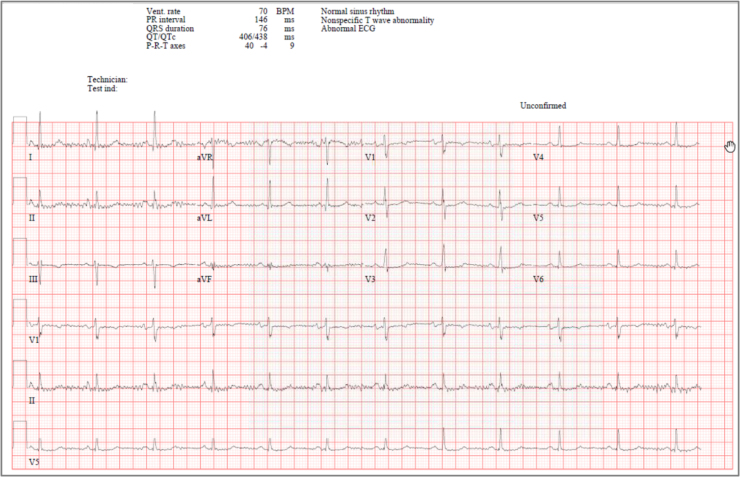
Baseline electrocardiogram for patient case #2 before starting Remdesivir therapy.

Upon presentation, she was vitally stable, afebrile (temperature 37.2 °C), heart rate 70 bpm, maintaining oxygen saturation of 100% on room air. Physical examination was unremarkable and her initial ECG was showing normal sinus rhythm (Fig. 4).

Her baseline blood investigations were as the following: WBC 2.40 g/dl (4.0–10.0 g/dl), hemoglobin 11.2 g/dl (12.0–15.5 g/dl), CRP 49 mg/dl (0–5 mg/dl), Lactate dehydrogenase (LDH) 229 U/L (135–214 U/L) and ferritin 545 ng/ml, ALT 12 U/L, AST 20 U/L, and serum creatinine 69 μmol/l with eGFR 82 ml/min/1.73 m^2^. Her chest X-ray showed right lower zone infiltrates so she was admitted as a case of mild COVID-19 pneumonia. She was managed as per the national COVID-19 treatment protocol with Favipiravir and Amoxicillin-clavulanic acid oral therapy. During her hospital stay, she continued to spike fever, so Amoxicillin-clavulanic acid was changed to Ampicillin-sulbactam intravenous (IV) therapy and septic work-up was sent, which eventually came as negative.

**Fig. 5:**
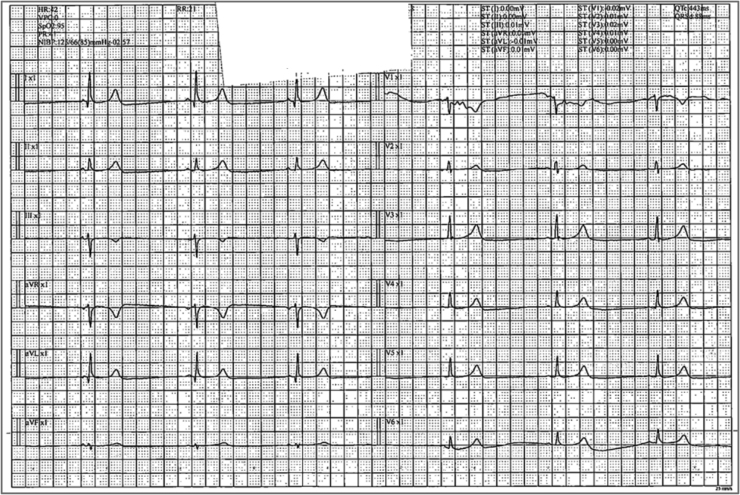
Electrocardiogram for patient case #2 few days after starting Remdesivir therapy.

Ten days later, the patient continued to spike fever, so Azithromycin was added. She eventually desaturated and she was requiring oxygen to maintain her saturation. Therefore, she was started on Remdesivir with a loading dose of 200 mg followed with 100 mg daily for four additional days to complete a total of 5-days course. She was also started on two units convalescent plasma and was started on Anakinra as her inflammatory marker was trending up. Her CRP increased to 89 and ferritin to 954, with a high interleukin-6 reading. Few days after starting Remdesivir, the patient was noted to be in sinus bradycardia with heart rate of 40–42bpm beats/min (baseline heart rate 60–70bpm beats/min) and further decreased to 37 bpm in the subsequent days (Fig. 5).

Further, her ECG was showing QTc interval prolongation with QTc interval of 518, so the Azithromycin was stopped as it can cause prolonged QTc interval. Few days later, the patient started to have symptomatic bradycardia (dizziness and fatigue), so she was shifted to Intensive Care Unit (ICU) for continuous cardiac monitoring. In the ICU, cardiologist was consulted, and she was started on atropine as needed (if bradycardia less than 35/min) and dopamine infusion 3 micro gram/min. Cardiac enzyme test results were negative and an echocardiogram was done for her and it was unremarkable. Holter monitoring was done as well and showed an episode of atrial fibrillation for 1 h and 42 min which was reverted by 150 mg of IV amiodarone.

Before receiving the third dose of Remdesivir, the decision was made to discontinue it. After that, her heart rate started to improve, ranging from 45 to 50 beat/min, over the subsequent days. The patient was discharged home after normalization of her heart rate to above 60 beat/min and her ECG was also normal (Fig. 3). Her renal and liver functions after Remdesivir therapy remained within normal ranges (ALT 11 U/L, AST 13 U/L, serum creatinine 59 μmol/l, eGFR 98 ml/min/1.73 m^2^). An echocardiogram was done later in the course assured normal motion motility, ejection fraction, and absence of any structural abnormality.

## Discussion

There is a growing body of evidence about the benefit of Remdesivir in severe COVID-19 pneumonia that lead to its extensive use during the COVID-19 pandemic. Subsequently, more side effects had been recognized in clinical practice. Previous studies during Ebola pandemic reported the incidence of cardiac side effects of Remdesivir including cardiac arrest, hypotension, bradycardia, and atrial fibrillation [10], and these effects have been also reported in COVID-19 studies [7], [8], [11], [12].

Limited data was available regarding bradycardia as an adverse effect of Remdesivir and only few cases were reported that were similar to our cases [13], [14], [15], [16]. One article presented two cases of Remdesivir-induced bradycardia, and one of these patients developed QTc interval prolongation as well. These adverse events were reverted upon the stopping of Remdesivir [13]. Another case report was describing a patient with a history of chronic left bundle branch block (LBBB), who developed bradycardia with symptoms of chest pain and dizziness and worsening of her baseline QRS duration in the ECG after starting Remdesivir. His symptoms were resolved after the discontinuation of Remdesivir and the administration of Atropine [14]. In a different case report of a 13-year-old child who received Remdesivir, bradycardia occurred after the initiation of Remdesivir therapy and heart rate was normalized 1 day after stopping it. Remdesivir [15]. A pharmacovigilance study was conducted to assess the association of the use of Remdesivir and the risk of reporting serious bradycardia. The results of the study revealed significant association between Remdesivir use and the incidence of bradycardia that was mostly serious, highlighting new safety concerns [17].

The possible mechanism for Remdesivir-induced bradycardia and cardiac toxicity could be due to the intrinsic electrophysiological properties that the endogenous nucleoside adenosine has. Being a nucleoside analog, Remdesivir was proposed to express a similar effect on the AV node, which theoretically may result in the observed cardiac dysfunction [18].

For the two case reports described here, patients did not have clear alternative explainations for the bradycardia as all other cardiac investigations including cardiac enzymes and the echocardiogram study in the second case failed to explain the new onset bradycardia. The possibility of drug-drug interaction was carefully checked and excluded. Both patients received Favipiravir prior to receiving Remdesivir therapy and Favipiravir may also induce bradycardia as reported in previous Ebolavirus and COVID-19 patients [19], [20]. Therefore, it is unknown whether the observed effect is due to a Remdesivir, Favipiravir, or an additive effect of the two drugs. Furthermore, patients with coronavirus disease may also develop bradycardia, thus it is unclear if these patients develop bradycardia due to the viral infection, antiviral therapy, or both [21]. However, the onset of bradycardia few days after the initiation of Remdesivir and the rapid restoration of regular cardiac rhythm and rate after its discontinuation pointed toward its possible effect.

In conclusion, the presented cases highlighted the potential cardiac adverse events and the incidence of sinus bradycardia with the use of Remdesivir therapy. Healthcare providers need to be aware of these events and to monitor the patients carefully to avoid serious – or even fatal – outcomes. Further large-scale cohort and case control studies are warranted for better understanding and correlation of Remdesivir use with cardiac adverse events.

## Consent

This case report was approved by the Institutional Review Board (MRC-04-21-224) and patients informed consents were obtained.

## CRediT authorship contribution statement

**AA**: Conceptualization, Writing – review & editing. **HM**: Data curation. **WO**: Data curation. **HE**: Data curation, Writing – original draft. **WM**: Writing – original draft. **MAA**: Resources, Writing – review & editing. **EZE**: Conceptualization, Writing – original draft.

## Role of funding source

The publication of this article was funded by the Qatar National Library. The funder had no role in the design of the study, interpretation of the data, writing the manuscript, or in the decision to submit the article for publication.

## Conflict of interest statement

All authors have seen and approved the final version of the manuscript being submitted, with no conflict of interest. They have no financial or personal interests or beliefs that could affect their objectivity of this work.
